# Supplementary material for: Reduction of non-typeable results using a plasmid oriented Lymfogranuloma venereum PCR for typing of Chlamydia trachomatis positive samples
Source: PLoS One. 2020 Jun 4;15(6):e0233990. doi: 10.1371/journal.pone.0233990 (PMC7271987; doi:10.1371/journal.pone.0233990)
Supplement: S1 File — (PDF) [file pone.0233990.s002.pdf]

Amsterdam UMC
Universitair Medische Centra

Aan mevrouw dr. S.M. Bruisten
Infectieziekten, GGD Amsterdam
Streeklaboratorium, k571, Nieuwe Achtergracht 100, 1018 WT Amsterdam

Medisch Ethische Toetsingscommissie AMC
XT4-148
telefoon: 020 56 67389

Amsterdam, 9 januari 2020
uw kenmerk:
ons kenmerk: W19_496 # 20.014
betreft:
Uw project: **Reduction of non-typeable results using a plasmid oriented Lymfogranuloma venereum PCR for typing of Chlamydia trachomatis positive samples**

Geachte mevrouw Bruisten,

Uw brief d.d. 18 december 2019 betreffende bovengenoemde studie is op 8 januari jl. besproken in de vergadering van het dagelijks bestuur.

Het dagelijks bestuur is van oordeel dat bovengenoemde studie niet valt binnen de reikwijdte van de Wet medisch-wetenschappelijk onderzoek met mensen, aangezien er geen sprake is van wetenschappelijk onderzoek zoals bedoeld in artikel 1, eerste lid onder b van de WMO, daar geen proefpersonen aan handelingen worden onderworpen bij gebruik van materiaal dat is verkregen tijdens normale diagnostiek.

Een formele beoordeling door onze commissie is derhalve niet noodzakelijk.

De commissie attendeert u op de volgende punten:
De commissie heeft alleen de WMO-plichtigheid beoordeeld. Er heeft verder geen inhoudelijke toets van het onderzoek plaatsgevonden. U en uw afdeling zijn verantwoordelijk voor de correcte uitvoering van het onderzoek volgens de geldende wet- en regelgeving. Hierbij vragen wij uw aandacht voor de belangrijkste regelgeving:

- Voor prospectief onderzoek, waarbij gegevens van proefpersonen worden verzameld en verwerkt, is toestemming van de proefpersonen nodig.
- Voor retrospectief onderzoek, waarbij gegevens van proefpersonen gecodeerd worden verzameld en verwerkt is in beginsel toestemming van de proefpersonen nodig. In artikel 458 van de WGBO is vastgelegd onder welke omstandigheden van het vragen van toestemming kan worden afgezien. Bij retrospectief *anoniem* onderzoek is toestemming niet verplicht, hierbij zijn de gegevens nooit meer herleidbaar tot de proefpersonen. Dus ook niet via een code.
- Wanneer in een onderzoek gegevens worden verzameld van proefpersonen, dient hiermee correct te worden omgegaan zoals bepaald in de Gedragscode Gezondheidsonderzoek (Code Goed Gedrag), Algemene Verordening Gegevensbescherming en de Uitvoeringswet Algemene Verordening Gegevensbescherming
- Wanneer in een onderzoek (lichaams)materiaal van proefpersonen wordt verzameld en verwerkt dient hiermee correct te worden omgegaan zoals bepaald in de Code Goed Gebruik.
- Onderzoek met anoniem materiaal vanuit de zorg is toegestaan, voorzover de patiënt van wie het materiaal afkomstig is hier geen bezwaar tegen heeft gemaakt (artikel 467 WGBO).

Meer informatie over bovengenoemde regelgeving kunt u vinden op internet.
Deze opsomming betreft de belangrijkste regelgeving, maar is niet uitputtend. Mogelijk is nog andere wet- en regelgeving van toepassing op uw onderzoek.

Indien u twijfelt of door amendering of het toevoegen van addenda het onderzoek nog steeds buiten de

reikwijdte van de WMO blijft kunt u dit aan de commissie ter beoordeling voorleggen.

Met vriendelijke groet,
namens de Medisch Ethische Toetsingscommissie AMC,

Mw.dr. C.L. van der Wilt
secretaris

Bijlage: verklaring in het Engels

c.c. per email: SmitP@maasstadziekenhuis.nl; pieterwsmit@gmail.com;
acornelissen@ggd.amsterdam.nl

English appendix

Medical Ethics Review Committee AMC
XT4-148
phone: +3120 56 67389

Amsterdam, January 9, 2020
your reference:
our reference: W19_496 # 20.014app.
subject:
Your project: **Reduction of non-typeable results using a plasmid oriented Lymfogranuloma venereum PCR for typing of Chlamydia trachomatis positive samples**

To whom it may concern,

Referring to our letter of January 9, 2020 (reference number W19_496 # 20.014) we are pleased to confirm that the Medical Research Involving Human Subjects Act (WMO) does not apply to the above mentioned study and that an official approval of this study by our committee is not required.

Yours sincerely,
on behalf of the Medical Ethics Review Committee of the Academic Medical Center,

Mrs C.L. van der Wilt, PhD
secretary
